# Dose and Exposure Time-Dependent Renal and Hepatic Effects of Intraperitoneally Administered Fumonisin B_1_ in Rats

**DOI:** 10.3390/toxins10110465

**Published:** 2018-11-09

**Authors:** András Szabó, Judit Szabó-Fodor, Mariam Kachlek, Miklós Mézes, Krisztián Balogh, Róbert Glávits, Omeralfaroug Ali, Yarsmin Yunus Zeebone, Melinda Kovács

**Affiliations:** 1 MTA-KE-SZIE Mycotoxins in the Food Chain Research Group, Kaposvár University, Guba S. u. 40., 7400 Kaposvár, Hungary; szabo.fodor.judit@gmail.com (J.S.-F.); mezes.miklos@mkk.szie.hu (M.M.); balogh.krisztian@mkk.szie.hu (K.B.); kovacs.melinda@ke.hu (M.K.); 2 Faculty of Agricultural and Environmental Sciences, Kaposvár University, Guba S. 40., 7400 Kaposvár, Hungary; mariamkachlek@gmail.com (M.K.); vomer2011@gmail.com (O.A.); yasminzeebone@yahoo.com (Y.Y.Z.); 3 Somogy County Kaposi Mór Teaching Hospital, Dr. József Baka Diagnostical, Oncoradiological, Research and Educational Center, Guba S. u. 40., 7400 Kaposvár, Hungary; 4 Faculty of Agricultural and Environmental Sciences, Department of Nutrition, Szent István University, Páter K. u. 1., 2013 Gödöllő, Hungary; 5 Autopsy Ltd., Telepes u. 42., 1147 Budapest, Hungary; glavits.robert.dr@gmail.com

**Keywords:** fumonisin B_1_, rat, dose-dependence, hepatotoxicity, nephrotoxicity, genotoxicity

## Abstract

Male Wistar rats were treated intraperitoneally (i.p.) with fumonisin B_1_ (FB_1_; 0, 20, 50 and 100 mg/kg dietary dose equivalent) for 5 and 10 days (*n* = 24–24 in each setting) to gain dose- and time-dependent effects on antioxidant status and oxidative stress response, clinical chemical endpoints and liver, kidney and lung histopathology and lymphocyte damage (genotoxicity). FB_1_ decreased feed intake, body weight gain and absolute liver weight, irrespective of the toxin dose. Relative kidney weight increased in the 10-day setting. Linear dose response was found for plasma aspartate aminotransferase, alanine aminotransferase, total cholesterol, urea and creatinine, and exposure time-dependence for plasma creatinine level. The latter was coupled with renal histopathological findings, tubular degeneration and necrosis and the detachment of tubular epithelial cells. The pronounced antioxidant response (reduced glutathione accretion, increasing glutathione peroxidase activity) referred to renal cortical response (5–10 days exposure at 50–100 ppm FB_1_). Hepatic alterations were moderate, referring to initial phase lipid peroxidation (exposure time dependent difference of conjugated diene and triene concentrations), and slight functional disturbance (↑ total cholesterol). Lymphocyte DNA damage was moderate, supporting a mild genotoxic effect of FB_1_.

## 1. Introduction

Fumonisins (FUMs) are mycotoxins produced predominantly by *Fusarium verticillioides* and *Fusarium proliferatum*. The 28 FUM analogues that have been characterized since 1988 can be divided into four main groups identified as fumonisin A, B, C, and P series [1]. Among the four groups (FA, FB, FC and FP), the toxicologically most important compounds are the B analogues. They are hydrocarbon long-chain aminopolyols with two tricarballylic acid side chains. Fumonisin B_1_ (FB_1_) is the most important representative of FBs [2]. In most cases, in field samples, FB_1_, fumonisin B_2_ (FB_2_) and fumonisin B_3_ (FB_3_) contributed to approximately 70%, 20% and 10% of FBs, respectively [2]. FB_1_ is generally found in the highest concentrations in maize and maize-based products [3,4].

A series of studies reported the occurrence of FB_1_ and FB_2_ in maize and products thereof in different European countries [5,6,7,8,9]. Although occurrence is widespread, concentration levels ranged only between 0.2 and 2 mg/kg, with generally higher levels in unprocessed materials.

In vivo studies on the toxicity of FB_1_ indicate that kidneys and liver are the main target organs. FB_1_ is the causative factor of equine leucoencephalomalacia [10], porcine pulmonary oedema syndrome [11] and hepatocellular carcinoma in rats [12]. FB_1_ is possibly carcinogenic for humans; it has been classified as a possible carcinogen (group 2B) [13]. All FUMs are highly polar and water soluble compounds. Structurally, the fumonisin backbone resembles the sphingoid bases sphinganine (Sa) and sphingosine (So) especially with the amino and hydroxy groups in positions C2 and C3. The key event is fumonisin-mediated inhibition of ceramide synthases (CerS). Inhibition of CerS results in the disruption of sphingolipid metabolism and, as a consequence, alterations in other lipid pathways. FUMs are regarded as structural analogues of free sphingoid bases and they competitively inhibit CerS, a group of key enzymes in the biosynthesis of ceramide and more complex sphingolipids [14].

Toxic substances generally negatively affect the overall health status of an organism, sometimes inducing direct oxidative stress. Focusing specifically on FB_1_, results gained so far on rodents suggest induction of oxidative stress directly or indirectly. In mice, in the spleen, exposure to the FB_1_ led to increased caspase-3 activity, lipid peroxidation, and interleukin-10 (IL) and IL-4 mRNA levels, but decreased reduced glutathione (GSH) content and down-regulated expression of glutathione peroxidase (GSPHx) and superoxide dismutase, and of interferon-γ and tumor-necrosis factor α mRNA [15]. In our earlier studies, high dose (50 ppm), but short exposure (5 days) in rats depleted hepatic GSH and induced lipid peroxidation (malondialdehyde, MDA), without activating the enzymatic defense [16]. In rabbits, the hepatic lipid peroxidation was minimal, as assessed by GSH, GSHPx and MDA, after feeding 10 ppm FB_1_ for 4 weeks [17]. The nature of FB_1_-induced oxidative stress is likewise acute. We were able to assess augmented lipid peroxidation in a short-term treatment [16], which was not present after prolonged FB_1_ exposure [17]. A typical form of this adaptation has already been reported [18] where, in vitro, primary hepatocytes provided increased membrane lipid levels of C18:1 n9 (oleic acid) and reduced polyunsaturated fatty acid (PUFA) niveau, as a means to provide selective resistance to FB_1_-induced oxidative stress, and even apoptosis resistance. Besides in vitro results, recent studies reported that the generation of reactive oxygen species (ROS) and oxidative stress was closely related to FB_1_s immunotoxic effect [19]. The most comprehensive review on the oxidative stress-mediated toxicity of FB_1_ has reported DNA damaging and genotoxic effects, as well as lipid peroxidation, all via a ROS-mediated manner [20]; however, as a limitation, most studies merely ascertained the toxic effect, but dose-response analysis is rare.

In this study, male Wistar rats were treated intraperitoneally (i.p.) with varying doses of FB_1_ (0, 20, 50 and 100 ppm dietary dose equivalent) over periods of 5 and 10 days to ascertain the dose- and time-dependent effect of FB_1_ in mediating oxidative stress.

## 2. Results

### 2.1. Feed Intake, Body and Organ Weights

All toxin-treated groups provided lower daily feed intake values from experimental days 3 and 4 onwards. Increasing exposure (20, 50, 100 ppm) decreased feed intake to 68, 54 and 42% until day 5 and to 51, 45 and 25% by day 10, respectively, as compared to the control. As a strongly relevant factor, the body weight (BW) gain showed a similar pattern in the control and treated groups, namely all toxin-treated groups showed BW loss, if expressed in a cumulative manner, for the 5 and the 10 experimental days (Table 1). The absolute BW at the end of the treatments was lower in all toxin-treated groups in the 5-day setting, as compared to the control, while below-control values were found for the 50 and the 100 ppm treatments in the 10-day study.

The absolute liver weight and, similarly, the relative liver weight was higher in the control animals in both treatments (5 and 10 days), as compared to all toxin-treated groups, irrespective of the dose (Table 1).

Comparing the exposure-time dependent differences, the group mean values between 5 and 10 days in case of absolute liver weight were different in the 50 and the 100 ppm treatments (Table 1, bold data pairs).

The absolute kidney weight was not providing dose-related differences in the 5-day treatment, while in the 10-day setting the 50 ppm group had significantly lower values as with the 100 ppm one. The relative kidney weight was the highest in the 100 ppm animals, as compared to the control and the 20 ppm in the 10-day treatment.

Kidney absolute and relative weight was different between the 5- and 10-day treatments in the 20 and the 100 ppm cases (Table 1, bold data pairs).

The absolute lung weight was lower in all toxin treated groups in the 5-day treatment, as compared to the control, while in the 10-day setting difference was not found. The relative lung weight of the control was significantly lower, as compared to the 100 ppm animals on day 10.

Comparing the 5- and 10-day treatments, the absolute lung weight was different in the control group, while the relative weight was different in the control, the 50 and the 100 ppm settings (Table 1).

### 2.2. Blood Clinical Chemistry

Blood plasma data determined, along with the inter-group differences are summarized in Table 2.

#### 2.2.1. Enzymes

The plasma aspartate aminotransferase (AST) and alanine aminotransferase (ALT) activities showed a toxin dose associated increase in both treatments (5 and 10 days). Both enzymes reached peak activity values in the 100 ppm treatment.

#### 2.2.2. Nitrogenous Metabolites

The plasma total protein concentration was the highest in the 50 ppm group in the 5-day treatment, while in the 10-day study there was no difference among the groups. Albumin showed an identical concentration pattern.

Comparing the 5- and 10-day treatments, total protein was lower in the 50 ppm treatment in the 10-day setting, as compared to the 5-day one (bold data pair).

Plasma creatinine concentration increased in parallel with the increasing mycotoxin dose, the highest plasma concentration was measured in the 100 ppm group, in both treatments. Comparing the exposure time-associated differences, the 10-day treatment induced significantly higher creatinine concentrations, as compared to the 5-day setting in case of 100 ppm.

Plasma urea concentration showed gradual increase in both treatments; the lowest concentrations were measured in the control, while in both treatments the 100 ppm treatment was associated with the highest plasma levels.

#### 2.2.3. Lipids

After the 5-day treatment total cholesterol concentration was only different (control < FUM) between the control and the 100 ppm groups, while in the 10-day treatment the control was different from all toxin-treated groups.

#### 2.2.4. Glucose

Regarding the plasma glucose level, a reduction was found to be associated with the toxin administration in both treatment periods (5 and 10 days). In contrast, the highest level was in the control from the two experiments, the 5-day treatment providing the higher values compared to the 10-day treatment (bold data pair).

#### 2.2.5. Dose-Dependence of the Plasma Clinical Chemical Compounds

From the enzymes, nitrogenous compounds and also from the lipids, datasets were tested for dose-dependent responses. The two treatments were handled separately. Table 3 summarizes the variables in which the linear regression model fitting was resulting R square values over 0.8. AST, ALT, total cholesterol, urea and creatinine were providing well-fitting linear response in accordance with the increasing toxin doses.

### 2.3. Antioxidant and Lipid Peroxidation Parameters

#### 2.3.1. Liver

In the hepatic concentration of reduced glutathione (GSH), conjugated trienes (CT) and conjugated dienes (CD), there was no significant difference among the groups (Table 4). The glutathione peroxidase (GSHPx) activity was the highest in the 50 ppm group after 5 days (being significantly higher as the value in the 10-day setting); a significant difference was only proven between the 20 and 50 ppm groups. The malondialdehyde (MDA) concentration was the lowest in both treatment periods in the control animals, and the highest levels were reached in the 20 ppm groups.

Meanwhile, the increasing mycotoxin concentration did not induce any change in the CD and CT concentrations, the 10-day treatment led to significantly higher concentrations in the 20 and the 50 ppm groups (5 vs. 10 days).

#### 2.3.2. Kidney

The renal concentration of GSH was significantly different from the control in the 100 ppm group (5 days). In the 10-day setting all toxin-treated groups provided higher GSH tissue concentrations than the control (Table 5).

In the 5- and 10-day treatments, in the case of the kidney, GSH differed between the control and the two 50 ppm groups.

The GSHPx activity showed a decrease in the 20 ppm group in the 5-day study (as compared to the 50 ppm), while in the 10-day study all toxin treated groups showed higher GSHPx activities, as compared to the control.

GSHPx activity was different between the 5- and 10-day treatments only at the 20 ppm dose.

The renal MDA concentration was the lowest in the 50 ppm group in both treatments; in the 5-day setting this differed from the 20 and the 100 ppm group means, while in the 10-day setting a difference was only proven between the 50 ppm and the control groups.

#### 2.3.3. Lung

In the lungs GSH and GSHPx were non-responsive. The only compound providing proven difference among groups was MDA. In the 5-day setting the 20 and the 50 ppm groups differed significantly, but a clear alteration pattern could not be seen. In the 10-day treatment the control differed from the 20 and the 100 ppm groups, with the control providing significantly lower values (Table 5).

The MDA concentration was significantly higher in the 10-day treatment, in all toxin fed groups, as compared to the 5-day treatment.

#### 2.3.4. Plasma

The plasma GSH concentration was not following any trend in the 5-day treatment, while in the 10-day treatment its plasma concentration was the highest in the 100 ppm group. The 5 and 10 days’ exposure differed at the 50 ppm level significantly (Table 5).

The plasma GSHPx activity decreased in the 5-day treatment in the 20 and the 50 ppm treatments, 20 ppm showing a difference between the 5- and 10-day protocols as well.

The plasma MDA concentration was the highest in the 100 ppm group (in both treatments, as compared to the control).

### 2.4. Genotoxicity

In both treatments (5 and 10 days) a dose-dependent effect was observed in tail intensity. In the 5-day exposure, a significant difference was observed among the control and the experimental groups whereas no difference was observed among the experimental groups. Similarly, in the 10-day exposure experiment all toxin treated groups differed significantly from the control. Moreover, tail intensity mean score of the 20 ppm group was significantly lower than that of the 100 ppm group. When applying a linear dose-response estimation, the accuracy of the two models was *R*^2^ = 0.81 and 0.97 in the 5 and 10 day settings, respectively.

Tail intensity (raw data-not shown) did not exceed 16% which is considered as low genotoxicity [22].

### 2.5. Histopathology

#### 2.5.1. Liver

**Figure 1:**
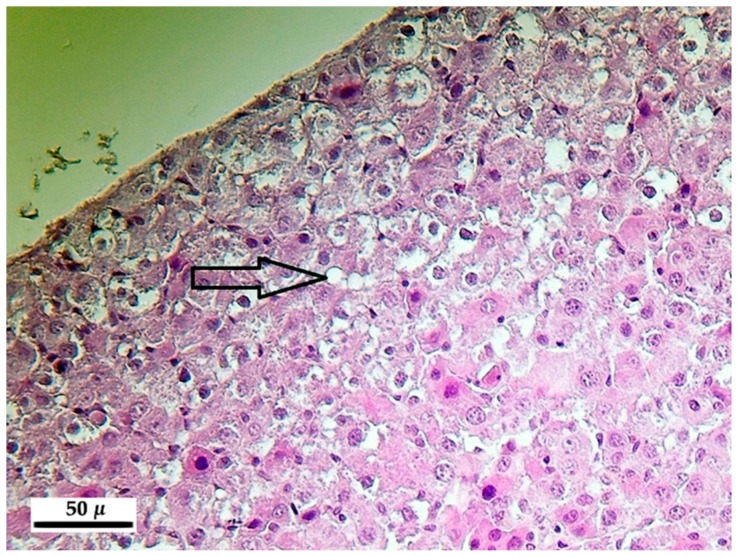
Vacuolar degeneration of the hepatocytes (arrow) after 100 ppm fumonisin B_1_ exposure in the 10-day experiment (hematoxylin-eosin, 400×).

**Figure 2:**
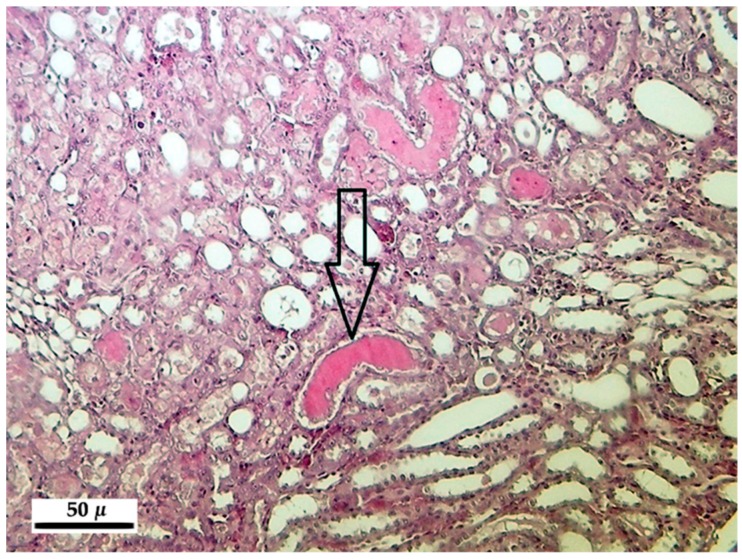
Solitaire necrosis and formation of Councilman bodies (arrow) in the liver of rats after 100 ppm fumonisin B_1_ exposure in the 10-day experiment (hematoxylin-eosin, 400×).

In animals of different groups and in different, non-systematically classified individuals the vacuolar degeneration of hepatocytes (Figure 1) was observed, along with a blurry pattern of the cytoplasm, in some cases with the disintegration of the cytoplasm, with casual fragmentation of it. In addition, hepatocytes with rounded shape occurred in a spread occurrence pattern; these were seceded from the regular cell organization lining and sometimes underwent necrosis (formation of Councilman bodies, Figure 2). These phenomena were observed in all three, toxin-treated groups (20, 50, 100 ppm). The occurrence frequency and the severity of these were increasing in parallel with the increasing toxin concentration and the length of exposure (Table 6). The above phenomena were matched with the proliferation of the cells belonging to the mononuclear phagocyte system (MPS) in the liver of animals exposed to 50 and 100 ppm, mostly occurring at the sites showing severe degenerative alterations.

#### 2.5.2. Kidney

**Figure 3:**
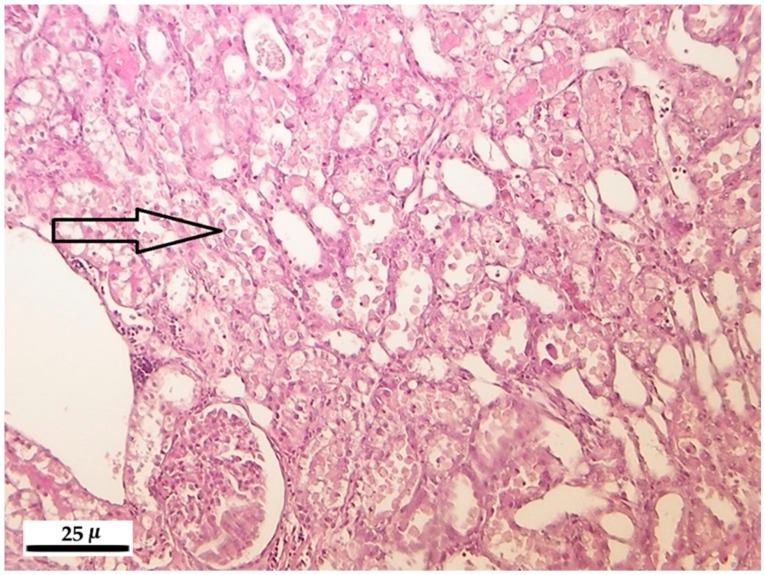
Necrotic and detached tubular epithelial cells in the tubular lumen (arrow) of the kidney after 100 ppm fumonisin B_1_ exposure in the 10-day experiment (hematoxylin-eosin, 200×).

**Figure 4:**
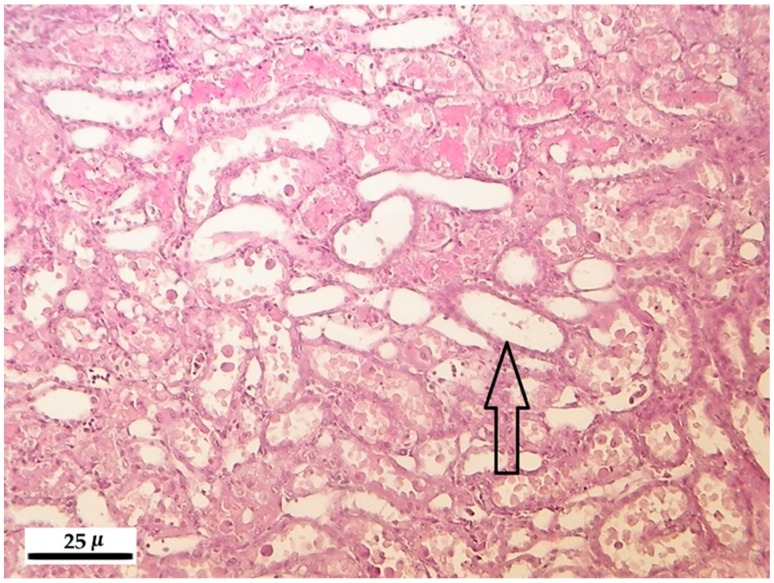
Tubular dilatation (arrow) in the kidney of rats after 100 ppm fumonisin B_1_ exposure in the 10-day experiment (hematoxylin-eosin, 200×).

In rats treated with different toxin concentrations for different time periods, the renal epithelial tubular cells showed degeneration, and in some instances their necrosis or detachment occurred (Figure 3). The slighter, first mentioned modification was characteristic to lower exposure, while the second symptoms were occurring in the 50 and 100 ppm exposed animals. In the group of rats exposed to 100 ppm for 10 days, in well-defined areas, the dilatation of tubule groups was found (Figure 4), sometimes the internal epithelium was atrophic or fully absent; in their lumen, hyalin cones were developed with typical eosinophil stain adsorption. The latter finding refers to the serious damage of the renal excretory function.

#### 2.5.3. Lung

Pulmonary oedema or histopathological alteration of the lung was found in none of the animals, irrespective of the treatment.

## 3. Discussion

The main target organ of FB_1_ toxicity in rats is the kidney, while in mice it is the liver (WHO 2001) [23]. Renal carcinogenicity of FB_1_ is more pronounced in male rats and liver carcinogenicity in female mice [24]. Accordingly, we tested male rats and analyzed both organs mentioned.

### 3.1. Feed Intake, Body and Organ Weights

In the study of Bondy et al. [25] body weight and feed intake values decreased as the result of i.p. FB_1_ treatment (0.75 mg/kg BW for 4 and 6 days), similarly to our study. The authors speculated that in their study possible dehydration occurred, since plasma albumin concentration increased. In our study, the increase of albumin level (similarly to total protein) was temporary and was observed only in case of the 50 ppm setting in the 5-day exposure. We also observed polyuria, but data were not recorded, and clinical chemistry results did not support dehydration (concentration of urea and creatinine increased but it could be attributable to the impaired renal function caused by the time- and dose-dependent renal toxicity of FB_1_). Thus, significantly decreased feed intake and hepato- and nephrotoxic effects may be, at least partly, contributors of the BW loss. Hepatic influencement is further supported by the increasing blood total cholesterol levels, in spite of decreasing BW and feed intake [25].

### 3.2. Serum Clinical Chemistry

#### 3.2.1. Enzymes

The AST and the ALT activities showed a toxin dose associated increase in both treatment periods. Elevated ALT and AST activity values were similarly observed when purified FB_1_ was administered i.p. to male Sprague–Dawley rats at 7.5 or 10 mg/kg body weight/day for 4 consecutive days [26]. Although AST is not merely liver specific, hepatic damage is sensitively and proportionally (in a linear manner, Table 3) indicated by it in case of FB_1_ toxicosis, irrespective of the length of exposure. Similar results were reported in piglets after 9 days and 1.5 mg/kg BW FB_1_ [27]. However, i.p. results on the clinical chemical composition of rat blood are rare in the literature. If gavage was used, Bondy et al. [28] reported increased ALT activity in the rat serum after 5 mg FB_1_/kg diet/day gavage dose. In one of our related studies, when gavaging rats with a 5 mg/kg diet FB_1_, we did not detect any alteration in the aminotransferase activities (Szabó et al. 2018; unpublished dataset), but ALT has been found to play a crucial role in shaping the total group variance, as analyzed by principal component analysis.

#### 3.2.2. Nitrogenous Metabolites

The primary toxic effect of FB_1_ concerns the kidneys in rodents [29], which is mostly relevant in rats. The nephrotoxic effect may be induced even prior detectable hepatotoxicity, when feeding 9 mg/kg dietary FB_1_ [29]. Only 234 and 484 mg/kg dietary FB_1_ was leading to the degeneration of the renal tubular epithelium in male Fisher rats in the study cited, adding that this was already an apoptotic process. Bondy et al. [30] exposed male rats to 1, 5, 15, 35 or 75 mg FB_1_/BW kg and determined blood urea N, without alterations, while at a direct mycotoxin load intravenously (1.25 mg/BW kg) an increase was found in blood urea [31]. In addition, urinanalysis revealed primarily transient effects [30], with concentration peaks at days 6–8, and after 12 days values returned to the baseline. It is thus a novel finding that in this study both treatments induced systematical linear dose response for plasma urea, irrespective of the exposure length.

A dose of 15 mg FB_1_/diet kg/day induced increased cytoplasmic vacuolation of adrenal cortex cells, indicating that the adrenals are also potential targets of FB_1_ [25]. When analyzing nephrotoxicity of FB_1_, Bondy et al. [25] described elevated creatinine concentrations in the experimental rats, as well as higher uric acid levels; former is consonant with our data. In an earlier i.p. study [32], 4 days on 10 mg/diet kg FB_1_ dose increased creatinine excretion, but this was paired with minimal histopathological alterations. Our results are generally similar to this, even at the highest FB_1_ level: no alteration was found of the high molecular weight proteins in the plasma (total protein and albumin), referring to an unaltered glomerular permeability. Although creatinine excretion has already been reported as a non-specific effect of FB_1_-induced nephrotoxicity, here we newly report as well exposure time dependence, since at 100 ppm at 5 and 10 days it led to significantly different plasma creatinine concentrations (Table 2).

#### 3.2.3. Lipids

After the 5-day treatment total cholesterol concentration was only different between the control and the 100 ppm groups, while in the 10-day treatment the control was different from all toxin-treated groups. Elevated total cholesterol, which is a typical response to FB_1_ [27,31] was as well observed at 5 mg FB_1_/diet kg/day and higher in rats [25]. Not only i.p. exposure, but as well different toxin administration types have been found to increase serum total cholesterol (feeding [33]; gavage [30]; single intravenous dose of 1.25 mg/kg BW [31]). The effect of fumonisin B_1_ on the cholesterol homeostasis may be multi-factorial, since sphingomyelin is a component of circulating lipoproteins and thus has an effect on the cellular cholesterol uptake [34], and ultimately on the cholesterol release of lipoproteins. A new addition to this has been published by Burger et al. [35], reporting that the originally asymmetrical hepatic subcellular (microsomal, mitochondrial and nuclear fractions) membrane loses its integrity as a key result of FB_1_. Not only are fatty acid metabolism and enzyme activity perturbation induced by FB_1_, but membrane microdomains are compositionally modified, typically depleted in cholesterol. This overall structural modification of the membrane rafts is coupled with the modification of the signaling mechanism, ultimately shifting the cell regulation towards apoptosis [35].

#### 3.2.4. Glucose

The FB_1_-induced lowering in plasma glucose level is a rare finding. Similar results have only been published for heifers in a 24-week study [36]. The most relevant findings to explain this may be two factors: first, decreasing BW and liver weight, along decreased feed intake, associated with higher FB_1_ doses, may lower the plasma glucose level. Second, FB_1_ is a well-known ceramide synthesis inhibitor, and as such, has a pro-apoptotic effect on skeletal muscles [37]. Since lipoapoptosis occurs in skeletal muscle myotubes, at least partially via de novo ceramide accumulation (typical FB_1_ effect), an inhibiting downstream apoptotic signaling augments glucose uptake, as proven in vitro.

### 3.3. Antioxidant and Lipid Peroxidation Parameters

#### 3.3.1. Liver

In the liver, only the ultimate lipid peroxidation product, MDA, increased, but the highest concentration values were attained at the 20 ppm exposure in both treatments. It is known that FB_1_ increases lipid peroxidation in rat liver and primary rat hepatocytes [38]. It is worth mentioning that in rats the primary target organ of FB_1_ is the kidney, and this is followed by the liver [39]. Since MDA results from end-phase lipid peroxidation, it seems that hydrogen free radicals attacked tissue lipids, as already published in an earlier study in rats [16]. Interestingly, a lower dose for a longer exposure period (10 ppm for 14 days) failed to induce MDA increment in rabbits [17], referring to a possible dose-dependent effect of FB_1_ onward in vivo lipid peroxidation. It is rather interesting that initiation-phase lipid peroxidation, as assessed by the determination of conjugated dienes and trienes, was not increased in the liver by any of the treatments. The real physiological background of this phenomenon remains unresolved, since root component (membrane lipid fatty acid) depletion is not a realistic case. Anyway, it is important to note that CD and CT concentrations were significantly higher in the 10-day setting, for both compounds, even though a dose-dependent increase was not present in this study. Summarizing the above findings, either hepatic effect was less pronounced of the hepatic defense was more profound, as compared to the kidney. The results unequivocally support the statement [39] that nephrotoxicity of FB_1_ is stronger than its hepatotoxic effect, even at high FB_1_ doses.

Based on the results of Abdellatef and Khalil [40], who applied oral exposure to 0, 50, 100 and 200 mg FB_1_/kg diet (equivalent to 0, 6, 12 and 24 mg/kg BW per day, respectively) to male Sprague–Dawley rats for 4 weeks, the lowest observed adverse effect level (LOAEL) for liver and kidney oxidative stress has been declared as 6 mg/kg BW/day, which is 50, 20 and 10 times the dose compared to ours (120, 300 and 600 μg/kg BW/day).

#### 3.3.2. Kidney

GSH (reduced glutathione) is a potent antioxidant, being present in the tissues in relatively high concentrations. When analyzing the kidney, a gradual and significant increase was shown when comparing the control and the 100 ppm treatment. This result seems to be contradictory, since GSH is generally depleted quickly (12–48 h in rats after a single intravenous dose [31]) by oxidative stimuli or agents, in an acute case (porcine kidney cells [41]; murine spleen [15]; 50 ppm FB_1_, rat liver [16]). However, its tissue level may as well increase (recover) if the reason is not acute oxidative stress, but an adaptation to prolonged stimulus [31]. When analyzing exposure time-dependence for GSH, there was no systematic difference between the 5 and 10 days settings; there was a defined increase in the tissue GSH concentration, but linear model fitting was not possible. We assume no clearly defined dose-dependent increase of renal cortical GSH concentration, at least not in the time-frame of this study.

Since reduced glutathione is partly regenerated by GSHPx, its increased activity either in tissues (isoform 2) or in plasma (isoform 3) refers to enhanced antioxidant defense capacity of the organism [42]. Thus, both in the 5 and in the 10-days treatments, but most expressed in the latter, GSHPx-aided enzymatic defense was evoked by the mycotoxin treatment in the renal cortex; this defense may be the part background of the enzymatic adaptation. It may be visible that enzymatic adaptation was more serious in the 10-day case, but inter-group difference (5 vs. 10 days) was not found. This is consonant with our earlier results, in which 5 days on 50 ppm FB_1_ was not yet activating the enzymatic antioxidant defense in rats [16].

Moreover, this protective effect may be seen in the concentration of kidney MDA. There was practically no systematic effect of the toxin treatment on the tissue lipid peroxidation end product, thus, marked lipid peroxidation was not proven. We suppose that this result may be as well associated with the exposure in a more complex manner, since FB_1_ strongly augments free radical production. Interestingly, an opposite result has been published by Domijan et al. [39], where very low FB_1_ doses (50 and 200 microg/kg BW dietary FB_1_) induced renal lipid peroxidation (MDA ↑) after 15 days in rats. Thus, it seems that the effect of FB_1_ on ROS over-production is a consequence rather than a mechanism of its toxicity.

#### 3.3.3. Lung

In the lung, the antioxidant system is very well developed, since lung epithelium is continuously exposed to an inhaled, high concentration of oxygen [43]. Thus, the lung is generally more potent at inhibiting the oxidative attack arising from its function. This has been proven by the full lack of inter-group differences in the GSH concentration and in the GSHPx activity. In contrast, end-phase lipid peroxidation was significantly higher in two (20 and 100 ppm) intoxicated groups’ lungs, as assessed with the tissue MDA concentration in the 10-day treatment. Interestingly, as reported by Petrache et al. [44] ceramide, a second messenger lipid, is a crucial mediator of alveolar, apoptotic destruction and the inhibition of enzymes controlling de novo ceramide synthesis prevents alveolar cell apoptosis and oxidative stress. Our results are likewise opposite. The explanation of this contradiction may be that there exist six isoforms of ceramide synthase, with tissue-specific expression in mammals [27]. The expression and even the level of transcripts of CerS1 and 4 have been published not to decrease, but to increase in the lung as a result of FB_1_ intoxication of piglets [27]. In the detailed study, the authors prove that lung tissue extract was able to increase stearoyl-dihydrocermide synthesis, adding FB_1_ can either inhibit or activate dihydroceramide synthesis depending on tissue type and mycotoxin dose.

#### 3.3.4. Blood Plasma

Blood plasma provided differential reactions in terms of GSH concentration on the oxidative stress, since in the plasma there is a specific GSH isoform present. Although our analysis was not isoform specific, it is visible (Table 5) that the 10-day exposure led a tendentious increase of plasma reduced glutathione concentration, the control and the most strongly toxic treatments providing significant difference. Although GHSPx was not in all cases reactive (no evoked enzymatic defense), the final lipid peroxidation product, MDA provided a very clearly increasing trend, with a higher slope in the 10-day treatment. Domijan et al. [39] reported similar results in the low-dose rat treatment, indicating that the time-demand of the increase of plasma MDA concentration is more than 2 days (ca. 5–7 days). Abel and Gelderblom [38] suggested that lipid peroxidation might be the consequence rather than the cause of the FB_1_ effect on the cell level; anyhow, at a 250 mg/kg dietary FB_1_ level authors described marked hepato- and as well subcellular oxidative damage in rats. The authors demonstrated that addition of alpha-tocopherol to primary hepatocyte cultures completely abolished the FB_1_-mediated increase in thiobarbituric acid reactive substances, but provided only partial protection from FB_1_ hepatotoxicity, indicating that lipid peroxidation may occur secondarily to FB_1_ cytotoxicity. We ultimately suppose that FB_1_-induced lipid peroxidation is reflected by multiple lipid-associated parameters in the blood plasma: partly by augmented hepatic lipoprotein secretion (increased total cholesterol concentration) and the increase of plasma malondialdehyde levels.

### 3.4. Genotoxicity

**Figure 5:**
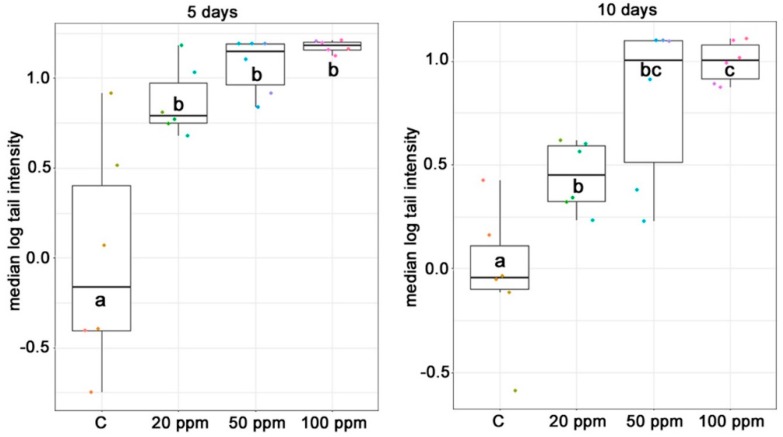
Log tail intensity values (median ± SD; *n* = 6/group) corresponding to DNA damage of lymphocytes after 5 and 10 days of intraperitoneal exposure of animals to increasing concentrations of FB_1_ (C: control, 20, 50, 100 mg/kg dietary dose equivalent).

Tail intensity (raw data not shown) did not exceed 16% in any of the treatments, which is considered to be low genotoxicity. This can be attributed to the short exposure period and it is comparable to previous results ([45]; Szabó-Fodor et al., 2018, unpublished observations). To the best of our knowledge there are no studies on the genotoxic effect (DNA damage) of FB_1_ on peripheral blood lymphocytes of rats. Although FB_1_ is a possible carcinogen [13], FB_1_ is considered as non-genotoxic [46]. According to Wang and Groopman [47], fumonisins are the only carcinogenic mycotoxins not having a direct DNA damage-inducing effect. Theumer et al. [48] investigated the genotoxic effect of dietary FB_1_ (100 mg/kg of feed) on male Wistar rats. Comet assay was used to assess the DNA damage in spleen mononuclear cells isolated from the rats. FB_1_ induced a very high tail intensity of 81.7 %. The high concentration of FB_1_ in combination with the prolonged exposure (90 days) could account for this high DNA damage. Although generally the extent of DNA damage was low, interestingly, the dose dependence could be characterized by a quasi linear relationship, as shown in Figure 5. The accuracy of fitting was higher in case of the longer treatment, and the scanning electron micrograph (SEM) of the dataset was visibly minor in the higher doses. This refers to a close relationship between dose, exposure time and the extent of damage.

### 3.5. Histopathological Analysis

#### 3.5.1. Liver

Following oral exposure to FB_1_, early signs of hepatotoxicity in rodents were apoptosis, necrosis, proliferation and regeneration, and hyperplasia of the bile duct. Histopathological alterations reported by Gelderblom et al. [49] in a longer rat-feeding trial of FB_1_ (26 months at 50 mg/kg diet FB_1_) appeared rather intense. Gelderblom et al. [49] observed that the liver of the rats killed from 12 months onwards appeared to be distorted and had a nodular appearance and a variegated color due to the presence of fatty changes, necrosis, hemorrhage and an irregular blood supply. In addition, the severity of lesions increased progressively towards the end of the experiment. Our results mostly support the onset of a necrotic process, adding that dose-associated severity has been observed: solitaire necrosis, vacuolar degeneration and cytoplasma fragmentation was more frequent at higher doses.

#### 3.5.2. Kidney

The kidney is the major target organ of FB_1_ in rats, but species-, strain-, and sex-dependent differences in dose response may occur [39]. In both liver and kidney, apoptosis alone or together with increased mitosis is an early microscopic indicator of tissue injury. Cytomegaly, anisocytosis, anisokaryosis, cytoplasmic vacuolation (hepatocellular) and necrosis may as well become evident in both tissues with increasing mycotoxin dose [50]. When purified FB_1_ was administered i.p. to the male Sprague–Dawley rats at 0.75 mg/kg BW/day for 2-4-6 consecutive days, Bondy et al. [26] observed minimal renal lesions with infrequent, typically single-cell necrosis, and desquamation of epithelial cells was observed. What we found was mostly the detachment of tubular epithelial cells at 50 ppm and over. This is consonant with findings in Spague–Dawley rats [32], with ezymuria as a result of tubular cell damage, using 7.5–10 mg FB_1_/BW kg intraperitoneally. The tubular degeneration found in our study is in agreement of the results of Suzuki et al. [32], reporting reduced anion transport in FB_1_-intoxicated rats. This result is further underpinned by the hyalin accumulation in our study, referring to compromised excretory function. Though the dose applied [32] was lower as in our treatment, authors also provided evidence of single cell necrosis at 10 ppm, which was not found in our present work. Higher dose (700 vs. 600 μg/kg BW) of FB_1_ (provided as fungal culture in the diet) and longer exposure (42 vs. 10 days) to ours did not affect the creatinine level in plasma, water intake, osmolarity and urinary excretion of sodium, while increased urine volume and potassium excretion and caused mild tubulointerstitial changes in the outer cortex of the kidney [51]. With regard to the histopathological alterations to the kidney in a long exposure, Gelderblom et al. [49] further described atrophic, pale, and the irregular outline and presence of numerous cortical and medullary retention cysts of the kidney.

#### 3.5.3. Lung

Pulmonary oedema or any other histopathological alteration of the lung was found in none of the animals, irrespective of the treatment. We conclude that pulmonary influencement was minimal at the exposure applied, meanwhile there exists a robust knowledge on the pulmonary effects of FB_1_ in pigs, but rats at the present exposure level are likely to be unaffected. The lung was analyzed based on our recent results according to which, during a 5-day FB_1_ feeding trial (50 ppm) the Hsp70 expression of the rat lung significantly increased by 40% as compared to the lung tissue of the control rats without significant changes in the antioxidant (GSH, GSHPx) and lipid peroxidation (MDA) parameters [52]. In the study of Salam et al. [53], male rats were fed 10 and 30 mg/kg BW FB_1_ in *Fusarium* culture material for 1, 4 and 8 weeks. Histopathology revealed an effect on the lung in the form of pulmonary congestion and alveolar edema, focal areas of interstitial edema, pulmonary congestion with inflammatory cellular infiltration already after 1 week. The severity and character of these pathological changes showed dose- and time-dependency.

## 4. Conclusions

Increasing FB_1_ dose and exposure interval was used to challenge the blood clinical chemical parameters, kidney, liver and lung antioxidant defense and histopathological response in male Wistar rats. We found a linear dose response for AST, ALT, total cholesterol, urea and creatinine in the plasma, and exposure time-dependence for the alteration of the plasma creatinine level. The latter parameter was coupled with marked renal histopathological alterations, tubular degeneration and necrosis, and tubular epithelial cell detachment. The hepatic oxidative stress response was moderate, mostly referring to initial phase lipid peroxidation (CD, CT), and slight functional disturbance (plasma total cholesterol increase). The lung was non-responsive for the treatment, while lymphocyte DNA damage was detectable, but moderate, supporting mild genotoxic effect of FB_1_.

## 5. Materials and Methods

### 5.1. Animals and Feeding

Adult, male Wistar Crl:WI BR rats (8 weeks of age at the beginning) were enrolled in the study and were kept in metabolic cages (Tecniplast, Castronno, Italy) individually. The animals (*n* = 6/group, total *n* = 48) were fed Ssniff R/M-Z+H feed (Ssniff GmbH, Soest, Germany). The rats were kept in a 12 h light and 12 h dark daily rhythm, at 20 °C in a rodent room, with a relative air humidity of 50%. Feed was offered ad libitum, and feed intake was measured daily.

Increasing concentrations of FB_1_ (0, 20, 50 and 100 mg/kg diet, expressed as feed dose equivalent and referred to as ppm in the entire text and tables) were tested in a short- and thereafter a long-term experiment (5 and 10 days, respectively), to assess dose and time associated toxic effects. The pure mycotoxin was purchased from Sigma-Aldrich (Schnelldorf, Germany), and stock solutions were prepared with sterile physiological salt solution. The solutions contained the daily toxin dose in exactly 1 mL, and this solution was administered as a single intraperitoneal dose. For the control animals (C), 1 mL of sterile physiological salt solution was dosed.

Mycotoxin treatment was set as follows: 36 µg/animal/day (approx. 120 µg/kg BW/day), 90 µg/animal/day (approx. 300 µg/kg BW/day) and 180 µg/animal/day (approx. 600 µg/kg bw/day). Calculating with the average feed intake of 30 g/animal/day and the absorption ratio of the toxin [54], the intraperitoneal (i.p.) administration represented the following dietary exposures: approx. 20, 50 and 100 mg/kg dietary equivalent for FB_1_.

The two, basically similar treatments lasted for 5 (*n* = 24) and 10 days (*n* = 24), respectively, only the exposure time being different. On days 6 and 11, after taking blood from the retro-orbital plexus, the animals were sacrificed by cervical dislocation and were immediately dissected.

### 5.2. Ethical Permission

The experimental protocol was authorized by the Food Chain Safety and Animal Health Directorate of the Somogy County Agricultural Office (Hungary), under the permission number SOI/31/00308-10/2017 (date of approval: 27 March 2017).

### 5.3. Clinical Chemical Parameters

The plasma total protein, albumin, creatinine, glucose, urea and the total cholesterol concentrations and the activity of aspartate aminotransferase (AST), alanine aminotransferase (ALT) were determined in a veterinary laboratory (Vet-Med Laboratory, Budapest, Hungary), using Roche Hitachi 917 Chemistry Analyzer (Hitachi, Tokyo, Japan) with commercial diagnostic kits (Diagnosticum LTD., Budapest, Hungary).

### 5.4. Antioxidant Status and Lipid Peroxidation

For the determination of lipid peroxidation, the samples of blood plasma, liver, kidney and lung were stored at −70 °C until analysis. Lipid peroxidation was determined by the quantification of malondialdehyde (MDA) levels with 2-thiobarbituric acid method in blood plasma and organ homogenates [55], and determination of conjugated dienes (CD) and trienes (CT) according to the AOAC [56] method in the liver. The tissue concentration of reduced glutathione (GSH) was measured by the method of Sedlak and Lindsay [57] and the activity of glutathione peroxidase (GSPHx) according to Lawrence and Burk [58].

### 5.5. Comet Assay of Lymphocytes

50 µL heparinized blood was added to 1 mL 1% low melting agarose gel in Eppendorf tubes on 37 °C. The suspension was mixed gently and two drops (140 µL) were transferred to slides previously coated with 1% normal melting point agarose. The slides were covered with cover slips and allowed to set. The cover slips were then removed and cell membranes were lysed with lysis buffer solution (1% Triton X-100, 2.5M NaCl, 10 mM Tris, 0.1 M EDTA, pH10) for 1 h at 4 °C. Following the lysis the slides were placed in alkaline electrophoresis buffer (pH 13) in an electrophoresis tank (Cleaver Scientific Ltd., Warwickshire, UK) for 40 min at 4 °C, followed by electrophoresis at 25 V (300 mA) for 30 min at 4 °C. The slides were then placed in neutralizing buffer (pH 7.5) and washed three times for 5 min, followed by a final wash in double distilled water for a further 5 min. The slides were then left to dry overnight and stained with ethidium bromide (30 µL) and covered with cover slips.

Tail intensity (TI, % DNA in the tail) was determined with an epifluorescent microscope (B600 TiFL; optimum filter 4 and λ = 302 nm) and Comet IV (version 4.3.1.) software (Perceptive Instruments Ltd., Bury St. Edmunds, UK), examining 50 comets per gel, 2 gels per slide, 1 slide per animal. A TI of zero corresponds to a cell with no DNA damage; increasing positive values of TI correspond to greater DNA damage to the respective cells.

### 5.6. Histopathological Analysis

After registering the macroscopic pathological signs on the internal and external organs, the liver, kidneys, lung and spleen were stored in 10% neutrally buffered formalin and were embedded into paraffin. For light microscopic analysis, microtome slides of 5 micron (µ) were prepared and stained with hematoxylin-eosin.

The main pathological alterations have been described and scored according their extent and severity as follows: 0 = no alteration, 1 = slight/small scale/few, 2 = medium degree/medium scale/medium number, 3 = pronounced/extensive/numerous.

The histopathological analysis was performed according to the Act #2011 (03.30) of the Hungarian Ministry of Agriculture and Rural Development and was in accordance with the ethical guidelines of the Organization for Economic Cooperation and Development (OECD) Good Laboratory Practice for Chemicals [59].

### 5.7. Statistical Analysis

Statistical analyses were performed using IBM SPSS 20.0 software (IBM Corp., Armonk, NY, USA) [60]. Data processing and the mathematical-statistical calculations were performed using the Compare Means (Independent-Samples-*t*-test) and Descriptive Statistics modules. Linear regression model was used to test dose dependent fitting in case of the blood biochemical parameters. Data were compared to reference range values for rats [21].

In the case of the comet assay, the experimental unit of exposure was the animal and statistical analysis was based on the individual animal response data. The constant of 0.001 was added to each TI before taking logarithms. The median values of 50 cells were calculated, and the average of the two medians (representing one gel = one animal) was used in the general linear model of one-way analysis of variance (ANOVA) as suggested by Bright et al. [61].

In the statistical analyses, differences between groups were considered significant when *p* values were <0.05.

## Figures and Tables

**Table 1:** Body weight (BW), absolute and relative organ weight of the rats in study (data are means ± standard deviation (SD) of 6 individuals/group).

Group	Days in Treatment	Control	20 ppm	50 ppm	100 ppm
BW_initial (g)	5	326.7 ± 15.2	323.6 ± 10.7	324.0 ± 9.60	331.1 ± 9.93
	10	325.5 ± 13.9	329.4 ± 6.9	336.9 ± 15.6	329.8 ± 20.6
BW_final (g)	5	**346.7 ± 14.6 ^b^**	316.9 ± 17.5 ^a^	315.5 ± 17.2 ^a^	308.1 ± 15.1 ^a^
	10	**368.0 ± 11.6 ^b^**	321.8 ± 28.5 ^ab^	283.4 ± 36.1 ^a^	270.7 ± 47.9 ^a^
BW_gain (g)	5	19.9 ± 2.29 ^c^	-6.73 ± 7.97 ^b^	−8.55 ± 7.95 ^b^	**−23 ± 10.7 ^a^**
	10	42.5 ± 13.4 ^c^	-7.58 ± 30.0 ^b^	−53.5 ± 29.0 ^ab^	**−59.1 ± 34.1 ^a^**
liver (g)	5	14.1 ± 1.18 ^b^	10.1 ± 1.37 ^a^	**10.1 ± 0.87 ^a^**	**8.97 ± 0.41 ^a^**
	10	15.2 ± 1.14 ^b^	9.12 ± 2.55 ^a^	**7.83 ± 1.66 ^a^**	**7.45 ± 1.41 ^a^**
rel. liver (%)	5	4.07 ± 0.28 ^b^	3.15 ± 0.29 ^a^	**3.22 ± 0.21 ^a^**	2.92 ± 0.08 ^a^
	10	4.12 ± 0.22 ^b^	2.92 ± 0.52 ^a^	**2.73 ± 0.32 ^a^**	2.80 ± 0.19 ^a^
kidney (g)	5	2.33 ± 0.19	**2.07 ± 0.12**	2.27 ± 0.27	**2.07 ± 0.16**
	10	2.47 ± 0.21 ^ab^	**2.42 ± 0.22 ^ab^**	2.30 ± 0.22 ^a^	**2.77 ± 0.39 ^b^**
rel. kidney (%)	5	0.68 ± 0.04	**0.63 ± 0.05**	0.72 ± 0.10	**0.67 ± 0.08**
	10	0.67 ± 0.05 ^a^	**0.78 ± 0.15 ^a^**	0.83 ± 0.10 ^ab^	**1.05 ± 0.21 ^b^**
lung (g)	5	**2.35 ± 0.28 ^b^**	1.82 ± 0.40 ^a^	1.60 ± 0.22 ^a^	1.52 ± 0.12 ^a^
	10	**1.73 ± 0.14**	1.60 ± 0.13	1.55 ± 0.14	1.58 ± 0.13
rel. lung. %	5	**0.68 ±0.07 ^b^**	0.57 ± 0.13 ^ab^	**0.51 ± 0.07 ^a^**	**0.49 ± 0.04 ^a^**
	10	**0.47 ± 0.04 ^a^**	0.52 ± 0.04 ^ab^	**0.55 ± 0.06 ^ab^**	**0.60 ± 0.07 ^b^**

^a, b^: small uppercase letters indicate significant difference (*p* < 0.05) between means of one row. Bold number pairs indicate significant difference (*p* < 0.05) between 5 and 10-day treatment data.

**Table 2:** Alterations in serum biochemical parameters study (data are means ± SD of 6 individuals/group) (AST: aspartate aminotransferase, ALT: alanine aminotransferase).

Serum Parameters	Days in Treatment	Control	20 ppm	50 ppm	100 ppm
AST (IU/L)	5	181.0 ± 19.8 ^a^	553.3 ± 116.2 ^b^	570.0 ± 54.0 ^b^	691.5 ± 189.7 ^b^
	10	170.5 ± 16.7 ^a^	406.0 ± 171.4 ^ab^	736.8 ± 432.8 ^bc^	1028.0 ± 418.0 ^c^
ALT (IU/L)	5	46.2 ± 5.27 ^a^	128 ± 36.72 ^b *^	208.8 ± 29.6 ^c *^	205.5 ± 52.7 ^c *^
	10	43.2 ± 4.17 ^a^	126.7 ± 29.47 ^ab*^	202.8 ± 102.5 ^bc*^	248.3 ± 81.3 ^c *^
Total protein (g/L)	5	57.0 ± 1.79 ^a^	58.2 ± 4.17 ^a^	**62.8 ± 1.92 ^b^**	59.3 ± 1.97 ^ab^
	10	58.8 ± 1.47	57.5 ± 3.62	**59.2 ± 2.93**	60.5 ± 1.76
Albumin (g/L)	5	32.7 ± 0.52 ^a^	33.2 ± 1.94 ^ab^	35.0 ± 1.22 ^b^	33.5 ± 1.05 ^ab^
	10	33.0 ± 0.63	33.5 ± 1.38	34.3 ± 1.63	34.0 ± 1.26
Glucose (mmol/L)	5	**8.87 ± 1.15 ^c^**	6.43 ± 1.92 ^b^	4.32 ± 0.61 ^a^	4.95 ± 0.64 ^ab^
	10	**7.15 ± 1.23 ^b^**	6.52 ± 2.25 ^ab^	4.13 ± 1.01 ^a^	4.55 ± 0.92 ^a^
Total Chol. (mmol/L)	5	2.60 ± 0.3 ^a^	4.63 ± 0.63 ^ab^	5.30 ± 0.45 ^ab^	6.43 ± 0.83 ^b^
	10	2.38 ± 0.15 ^a^	4.43 ± 0.52 ^b^	5.18 ± 0.91^b^	5.92 ± 1.58 ^b^
Urea (mmol/L)	5	8.85 ± 0.53 ^a^	10.1 ± 1.62 ^a *^	10.5 ± 0.86 ^ab *^	12.7 ± 2.11 ^b *^
	10	8.42 ± 0.35 ^a^	12.2 ± 5.18 ^a *^	13.0 ± 5.12 ^ab *^	22.9 ± 11.1 ^b *^
Creatinine (micromol/L)	5	30.3 ± 4.23 ^a^	47.2 ± 8.3 ^b^	57.0 ± 7.78 ^bc *^	64.5 ± 7.4 ^c *^
	10	26.3 ± 1.63 ^a^	56.2 ± 12.6 ^b *^	60.7 ± 9.03 ^bc *^	81.5 ± 25.1 ^c *^

^a, b^: small uppercase letters indicate significant difference (*p* < 0.05) between means of one row. Bold number pairs indicate significant difference (*p* < 0.05) between 5- and 10-day treatment data. * values above the physiological upper level, 24–49 IU/L for ALT, 4.0–9.3 mmol/L for urea and 31–48 µmol/L for creatinine according to Boehm et al. [21].

**Table 3:** Linear regression equation parameters in the two treatments.

Parameters	5 Days			10 Days		
	Slope	Constant	*R* ^2^	Slope	Constant	*R* ^2^
AST	154.8	11.9	0.82	290.3	−140.5	0.996
ALT	55.9	7.42	0.88	69.2	17.7	0.984
total_chol.	1.22	1.7	0.95	1.13	1.64	0.93
urea	1.19	7.58	0.93	4.44	3.04	0.86
creatinine	11.2	21.7	0.97	17	13.7	0.93

**Table 4:** Hepatic antioxidant and oxidation product data of the rats (data are means ± SD of 6 individuals/group) (GSH: reduced glutathione, GSHPx: glutathione peroxidase, CD: conjugated dienes, CT: conjugated trienes, MDA: malondialdehyde).

Liver	Days in Treatment	Control	20 ppm	50 ppm	100 ppm
GSH (micromol/g)	5	2.72 ± 0.76	3.01 ± 0.41	3.06 ± 0.71	2.95 ± 1.15
	10	3.2 ± 0.27	4.12 ± 1.19	3.52 ± 1	3.98 ± 1.14
GSHPx (IU/g prot.)	5	1.39 ± 0.31 ^ab^	1.11 ± 0.1 ^a^	**1.82 ± 0.42 ^b^**	1.6 ± 0.36 ^ab^
	10	1.42 ± 0.2	2.01 ± 1.02	**1.05 ± 0.53**	1.61 ± 1.06
MDA (nmol/g)	5	55.6 ± 3.19 ^a^	69.9 ± 4.09 ^b^	64.9 ± 7.57^ab^	68.9 ± 8.52 ^b^
	10	47.3 ± 14.2 ^a^	74.7 ± 12.4 ^b^	61.5 ± 13.2^ab^	63.6 ± 3.94 ^ab^
CD (Abs. 232 nm)	5	0.57 ± 0.02	**0.58 ± 0.03**	**0.59 ± 0.02**	0.56 ± 0.04
	10	0.6 ± 0.03	**0.62 ± 0.02**	**0.63 ± 0.04**	0.64 ± 0.12
CT (Abs. 268 nm)	5	0.22 ± 0.01	**0.21 ± 0.01**	**0.22 ± 0.01**	0.21 ± 0.01
	10	0.22 ± 0.01	**0.23 ± 0.01**	**0.24 ± 0.01**	0.25 ± 0.07

^a, b^: small uppercase letters indicate significant difference (*p* < 0.05) between means of one row. Bold number pairs indicate significant difference (*p* < 0.05) between 5- and 10-day treatment data.

**Table 5:** Kidney, lung and plasma antioxidant and oxidation product data of the rats (GSH: reduced glutathione, GSHPx: glutathione peroxidase, CD: conjugated dienes, CT: conjugated trienes, MDA: malondialdehyde).

**Kidney**	**Days in Treatment**	**Control**	**20 ppm**	**50 ppm**	**100 ppm**
GSH (micromol/g)	5	**2.65 ± 0.44 ^a^**	4.26 ± 1.09 ^ab^	**6.65 ± 0.79 ^ab^**	5.24 ± 1.00 ^b^
	10	**3.19 ± 0.47 ^a^**	4.68 ± 0.21 ^b^	**4.86 ± 0.46 ^b^**	5.3 ± 0.61 ^b^
GSHPx (IU/g prot.)	5	2.47 ± 0.38 ^ab^	**1.99 ± 0.86 ^a^**	3.13 ± 0.4 ^b^	2.5 ± 0.33 ^ab^
	10	2.11 ± 0.1 ^a^	**2.89 ± 0.35 ^b^**	2.82 ± 0.18 ^b^	2.97 ± 0.51 ^b^
MDA (nmol/g)	5	52.2 ± 8.0 ^ab^	57.7 ± 5.09 ^b^	44.3 ± 2.89 ^a^	63.4 ± 10.1 ^b^
	10	66.5 ± 11.3 ^b^	55.0 ± 8.27 ^ab^	41.9 ± 6.24 ^a^	54.8 ± 9.75 ^ab^
**Lung**	**Days in Treatment**	**Control**	**20 ppm**	**50 ppm**	**100 ppm**
GSH (micromol/g)	5	3.44 ± 0.39	3.81 ± 0.54	3.4 ± 0.37	3.41 ± 0.33
	10	3.49 ± 0.45	3.75 ± 0.47	2.94 ± 0.44	3.26 ± 0.58
GSHPx (IU/g prot.)	5	2.97 ± 0.42	3.26 ± 0.68	**3.16 ± 0.48**	3.06 ± 0.36
	10	2.69 ± 0.29	2.97 ± 0.63	**2.56 ± 0.27**	2.67 ± 0.48
MDA (nmol/g)	5	28.6 ± 6.54 ^ab^	**35.2 ± 6.67 ^b^**	**24.1 ± 3.16 ^a^**	**27.7 ± 4.28 ^ab^**
	10	35.3 ± 4.91 ^a^	**55.6 ± 5.03 ^c^**	**39.1 ± 2.78 ^ab^**	**42.9 ± 4.25 ^b^**
**Plasma**	**Days in Treatment**	**Control**	**20 ppm**	**50 ppm**	**100 ppm**
GSH (micromol/g)	5	3.34 ± 0.29 ^ab^	3.44 ± 0.34 ^b^	**2.8 ± 0.34 ^a^**	3.45 ± 0.38 ^b^
	10	3.16 ± 0.25 ^a^	3.46 ± 0.29 ^ab^	**3.32 ± 0.23 ^ab^**	3.97 ± 0.83 ^b^
GSHPx (IU/g prot.)	5	3.02 ± 0.35 ^b^	**2.47 ± 0.17 ^a^**	2.47 ± 0.36 ^a^	2.57 ± 0.33 ^ab^
	10	2.69 ± 0.25	**2.73 ± 0.19**	2.83 ± 0.29	2.58 ± 0.34
MDA (nmol/g)	5	12.4 ± 1.43 ^a^	14.1 ± 1.46 ^ab^	**14.5 ± 1.3 ^ab^**	15.3 ± 1.24 ^b^
	10	12.4 ± 0.91 ^a^	14.5 ± 1.63 ^b^	**12.4 ± 0.6 ^a^**	16.7 ± 1.11 ^c^

^a, b^: small uppercase letters indicate significant difference (*p* < 0.05) between means of one row. Bold number pairs indicate significant difference (*p* < 0.05) between 5- and 10-day treatment data.

**Table 6:** Time and dose dependent histopathological alterations in the liver, kidney and lung (*n* = 6/group).

	Number of Animals Showing the Respected Alteration in the Respective Group (*n* = 6/Group)								Total Score (Representing the Severity of the Alteration) ^1^							
Group (ppm)	0 (control)		20		50		100		0 (control)		20		50		100	
length of exposure (day)	5	10	5	10	5	10	5	10	5	10	5	10	5	10	5	10
Liver																
Vacuolar degeneration	0	0	6	6	6	6	6	6	0	0	6	12	9	14	13	16
Cytoplasma fragmentation	0	0	4	6	6	6	6	6	0	0	4	6	8	14	12	18
Solitaire necrosis (Councilman bodies)	0	0	2	3	4	6	6	6	0	0	2	3	4	12	10	18
MPS cell proliferation	0	0	0	0	0	4	3	6	0	0	0	0	0	4	30	12
Kidney																
Tubular degeneration	0	0	4	6	5	6	6	6	0	0	4	10	6	13	12	17
Tubular necrosis	0	0	0	0	0	6	1	6	0	0	0	0	0	8	1	12
Detachment of tubular epithelial cells	0	0	0	0	2	6	6	6	0	0	0	0	2	6	6	12
Tubular dilatation	0	0	0	0	0	1	0	6	0	0	0	0	0	1	0	15
Hyaline accumulation	0	0	0	0	0	0	0	6	0	0	0	0	0	0	0	11
Lung																
Any alteration	0	0	0	0	0	0	0	0	0	0	0	0	0	0	0	0

^1^ total score has been calculated as the sum of the individual scores (*n* = 6).
